# Lockdowns, lives and livelihoods: the impact of COVID-19 and public health responses to conflict affected populations - a remote qualitative study in Baidoa and Mogadishu, Somalia

**DOI:** 10.1186/s13031-021-00382-5

**Published:** 2021-06-12

**Authors:** Dorien H. Braam, Sharath Srinivasan, Luke Church, Zakaria Sheikh, Freya L. Jephcott, Salome Bukachi

**Affiliations:** 1 Disease Dynamics Unit, Department of Veterinary Medicine, University of Cambridge, Cambridge, UK; 2 Department of Politics and International Studies, University of Cambridge, Cambridge, UK; 3 Department of Computer Science and Technology, University of Cambridge, Cambridge, UK; 4 Africa’s Voices Foundation, Nairobi, Kenya; 5 Institute of Anthropology, Gender and African Studies, University of Nairobi, Nairobi, Kenya

**Keywords:** COVID-19, Coronavirus, Forced migration, Internal displacement, Humanitarian emergency, Somalia, Mogadishu, Baidoa, Conflict, Public health

## Abstract

**Background:**

Authorities in Somalia responded with drastic measures after the first confirmed COVID-19 case in mid-March 2020, closing borders, schools, limiting travel and prohibiting most group functions. However, the impact of the pandemic in Somalia thereafter remained unclear. This study employs a novel remote qualitative research method in a conflict-affected setting to look at how some of the most at-risk internally displaced and host populations were impacted by COVID-19, what determined their responses, and how this affected their health and socio-economic vulnerability.

**Methods:**

We conducted a remote qualitative study, using Katikati, a 1-to-1 conversation management and analysis platform using short message service (SMS) developed by Lark Systems with Africa’s Voices Foundation (AVF), for semi-structured interviews over three months with participants in Mogadishu and Baidoa. We recruited a gender balanced cohort across age groups, and used an analytical framework on the social determinants of health for a narrative analysis on major themes discussed, triangulating data with existing peer-reviewed and grey literature.

**Results:**

The remote research approach demonstrated efficacy in sustaining trusted and meaningful conversations for gathering qualitative data from hard-to-reach conflict-affected communities. The major themes discussed by the 35 participants included health, livelihoods and education. Two participants contracted the disease, while others reported family or community members affected by COVID-19. Almost all participants faced a loss of income and/or education, primarily as a result of the strict public health measures. Some of those who were heavily affected economically but did not directly experienced disease, denied the pandemic. Religion played an important role in participants’ beliefs in protection against and salvation from the disease. As lockdowns were lifted in August 2020, many believed the pandemic to be over.

**Conclusions:**

While the official COVID-19 burden has remained relatively low in Somalia, the impact to people’s daily lives, income and livelihoods due to public health responses, has been significant. Participants describe those ‘secondary’ outcomes as the main impact of the pandemic, serving as a stark reminder of the need to broaden the public health response beyond disease prevention to include social and economic interventions to decrease people’s vulnerability to future shocks.

## Background

### Pandemic

By October 2020, the total number of confirmed COVID-19 cases surpassed 40 million worldwide, with over one million fatalities, primarily affecting the United States, India and Brazil, while many countries relatively unaffected during the ‘first wave’ experienced dramatic increases in cases (WP, 20 Oct; WHO, 21 Oct). Early on, global health experts warned of the potential for devastating COVID-19 outbreaks in low- and middle-income settings (LMIS) due to a lack of availability, inadequate capacity and poor access of healthcare systems, however confirmed caseloads across the African continent have remained relatively low, including in Somalia.

For decades, Somalia’s status as a fragile and conflict-affected state has corresponded with weak health systems and poor health outcomes for the majority of its population. Somalia, with an estimated population of 15.9 million, consists of five federal states and its capital Mogadishu in Benadir province under federal authority, with varied levels of economic development depending on the states’ level of political stability and security [1]. Two of the country’s main economic sectors of livestock trade and remittances were negatively affected by the pandemic, including as a result of movement restrictions to control the spread of the virus [2]. The country is among the poorest countries in the Horn of Africa and designated as a ‘least developed country’ by the United Nations (UN), with its economic development affected by protracted crises causing widespread destruction and displacement, all further complicated by the pandemic [2]. With almost 70 % of the population living in poverty, by mid-2020 almost a third of the population was in need of humanitarian assistance due to crisis including floods, droughts, locusts invasions and COVID-19 [1]. The pandemic was projected to reduce the expected economic growth from 3.2 % to a negative 2.5 % in 2020, while inflation increased due to nationwide price increases [3].

The country lacks comprehensive health services, healthcare professionals and infrastructure, alongside unequal distribution of facilities and resources, means that many people lack access to healthcare altogether [4]. This is reflected in the World Health Organization (WHO) key health indicators for Somalia, which are among the lowest in the world, including high neonatal and maternal mortality rates, and a low life expectancy of 56.5 years [5]. In response to COVID-19, the Federal Government of Somalia pledged US$5 million towards a healthcare response fund, to develop and rehabilitate nationwide healthcare facilities [3]. However, the lack of current facilities means that COVID-19 testing is limited to suspected cases, thereby likely underestimating the burden of disease, with most testing taking place in the main urban centers [6].

The low case numbers in LMIS have to a large extent been attributed to limited testing capacities, exacerbated in fragile and conflict-affected countries such as Somalia [7]. Some researchers argue however, that the rapid government responses, fewer (international) travel movements, limited urbanization and a relatively young demographic in LMIS has limited infection rates, as morbidity and mortality rates are associated with older age [8, 9]. Modelling studies assessing the potential impact of COVID-19 in LMIS acknowledge significant uncertainties, due to a lack of primary data on transmission and health outcomes [8, 10].

Due to a lack of access, including through COVID-19 related movement restrictions and the ongoing complex humanitarian emergency, there has been little evidence on the actual status of the pandemic in Somalia. The proportion of confirmed positive tests remained fairly stable throughout March – October, 2020. Meanwhile, WHO data shows a varied picture across the country, which makes it difficult to draw conclusions [11].

Somali authorities implemented rapid and drastic measures to curb the spread of the pandemic in a challenging environment. As soon as Somalia identified its first COVID-19 cases in mid-March, the Federal Government established a national response committee and an incident management system. At the onset of the pandemic, Somalia had no laboratory capacity to diagnose the disease, and screening started with temperature checks at airports and isolation of suspected cases [6]. The Ministry of Health established a multisectoral emergency task force, deployed health workers at airports, and established isolation facilities for those arriving from high-risk countries [12]. Subsequently border crossings were closed and in-country movements restricted, while isolation center- and critical care capacity was increased [13]. The Ministry further developed a National Preparedness and Response Plan and Risk communication and community engagement (RCCE) strategies and taskforce, supported by national and international relief organizations [12, 14]. RCCE strategies through formal and informal channels initiated to prevent and control disease spread included the provision of a toll-free number for general advice, the use of radio, television and social media for mass health communications, with a focus on social distancing and hygiene [15]. Community Health Workers (CHW) conducted outreach, visiting communities to identify cases based on syndromic surveillance, tracing contacts and raising awareness [16].

By late September 2020, Somalia had confirmed 3588 COVID-19 cases, with 99 fatalities, while the utilization rate of isolation facilities remained low at 17 % [17]. The impact of the disease is difficult to investigate and assess however, not only due to a limited testing capacity, but also a lack of access to hard-to-reach populations in the ongoing complex humanitarian emergency, in particular during COVID-19, resulting in gaps in epidemiological trend data [18]. As a result of the protracted emergency, malnutrition rates are high and health outcomes poor, exacerbated by the lack of health services, which puts people at increased risk of infectious diseases, including COVID-19 [19]. Meanwhile, experts warned about the economic impact of curfews and lockdowns, control measures developed based on middle- and high-income contexts, potentially unsuitable to the local population [20].

The contribution of the pandemic to the existing barriers to access to populations in need poses an ongoing challenge for ensuring an effective response grounded in community experiences and priorities. Through this study, we deploy a novel remote qualitative research approach suited to fragile and conflict affected settings to gain a rapid grounded overview of how the disease and formal intervention measures impacted internally displaced and host populations, and how policy measures, local context and community responses influenced disease transmission, social and economic vulnerabilities.

### Internal displacement

Currently, there are an estimated 2.6 million Internally Displaced Persons (IDPs) in Somalia (IOM, 2020), primarily displaced due to the impact of floods or drought (72 %) and conflict (25 %) [21]. The most recent displacement was caused by severe flooding affected the southern regions, with over 650,000 newly displaced since June 2020. One of our participants was recently displaced due to droughts and locusts destroying crops and agricultural land. Internally displaced persons (IDPs) often depend on daily wages and have limited or no access to health facilities. Acute watery diarrhea, including suspected cholera, and measles are regularly reported in clinics serving IDPs [22].

IDPs in informal camps were considered most at risk of COVID-19, due to continuous in- and out movements and low-quality shelters [23]. Displaced populations often live in marginalized areas, in substandard and crowded living conditions in camps or slum settings, lacking sanitation and access to public health and social services, which puts them at higher risk of infectious diseases, including syndemic health risks such as malnutrition and underlying conditions, which often remain untreated [24, 25].

One of the main challenges of responders remains restricted humanitarian access due to ongoing insecurity, in particular in south and central Somalia. Refugees and internally displaced people (IDPs) further increase the pressure on limited health system capacity, with people often relying on private services when resources are available, or those provided by non-profit organizations [4]. People relying on services provided by relief agencies, such as health supplies, food and cash distributions, are likely to be greatly impacted by movement restrictions. Humanitarian responders therefore rapidly drafted plans to control COVID-19, focusing on strengthening of health systems, provision of protective equipment and RCCE. International and national nonprofit organizations provided training on COVID-19 surveillance, case management and RCCE, increased investigation and testing capacity, established health and (underutilized) isolation facilities. Community Health Workers (CHW) conducted outreach, visiting communities to identify cases, tracing contacts and raising awareness (WHO, August 2020). Nonetheless a first case was confirmed in an IDP camp on 28 April [26]. Our study takes a closer look at the experiences of these hard-to-reach populations during the early months of the pandemic.

### Social determinants of health

Disease infection and transmission does not only depend on the presence of pathogens, but on complex interactions of biomedical, environmental, socio-economic and political factors [27]. Socio-economic and health inequalities related to political and economic processes increase disease risks of resource-poor communities, especially those in countries with limited resources facing complex emergencies and limited healthcare [28]. Globally, COVID-19 has made these health inequalities even more visible. Pre-existing poor health conditions, which put people more at risk of the disease, may be caused or exacerbated by crowded, poor living conditions and a lack of sanitation, which characterize IDP camps [29]. These factors play a role in the risk of severe COVID-19, the ability to adhere to preventive measures, and the impact of public health approaches to lives and livelihoods. We therefore analyze our findings using a conceptual framework of social determinants of health developed by Solar and Irwin [30] for the World Health Organization (WHO), which explicitly aims to not only guide empirical work, but also influence policy making [30]. The strength of the framework lies in its inclusion of structural drivers of the social determinants, with political context particularly relevant in considering the impact of COVID-19 policies.

The aim of our study was to capture the burden of COVID-19 disease and socio-economic impact in households and communities on which there is limited data available, explore responses to the disease and increase understanding how these may influence vulnerability and wider determinants of health. We hope that this study and the learnings on the remote research method and tools that it employed will inform responses to the COVID-19 pandemic as well as other infectious diseases in vulnerable hard-to-reach populations.

## Methods

### Study design and setting

We used a remote web-based conversation and analysis platform, Katikati, to open, sustain and analyse 1-to-1 SMS text message interactions with research subjects over an extended period. This allowed us to capture qualitative interview data on the impact of COVID-19 to Internally Displaced Persons (IDPs) and host populations, including on their health status, responses to the disease, protective practices and what determined their response. Sending and receiving SMS was free for respondents. Through this method, we were able to capture the voices of participants at a time where travel restrictions and insecurity limit physical access to hard-to-reach populations. We conducted our study in Baidoa, the capital of Somalia’s Southwest state, and Mogadishu, the federal capital in Benadir province, which are among the largest urban centers in the country and host to respectively 246,000 and 497,000 IDPs, the largest such populations in Somalia. We contacted IDPs and host populations in Daynile district, Mogadishu and Baidoa, two places where IDPs are considered most at risk of COVID-19, based on indicators developed by IOM, related to the site location and size, frequency of new arrivals, shelter space and type, access to water and health support, and available information on humanitarian services, affecting infectious disease risk and options for disease prevention [31].

### Materials and participants

The study utilized a qualitative longitudinal case study approach, that recruited research subjects from audiences who had engaged with AVF’s COVID-19 RCCE interactive radio and SMS programming. To design the questionnaire, we used existing data on Somali IDPs, the COVID-19 response and WHO and CDC information on COVID-19 symptoms and transmission, as well as previously published studies on infectious diseases and epidemics in resource-poor settings. Following basic demographic questions to confirm people’s status as displaced or host population, we started with an open-ended question on how lives were impacted by COVID-19 to determine the focus of the conversation. Subsequently, we personalized the conversations dependent on participants’ responses, discussing themes including disease prevention, responses and health systems and socio-economic impact.

**Fig. 1:**
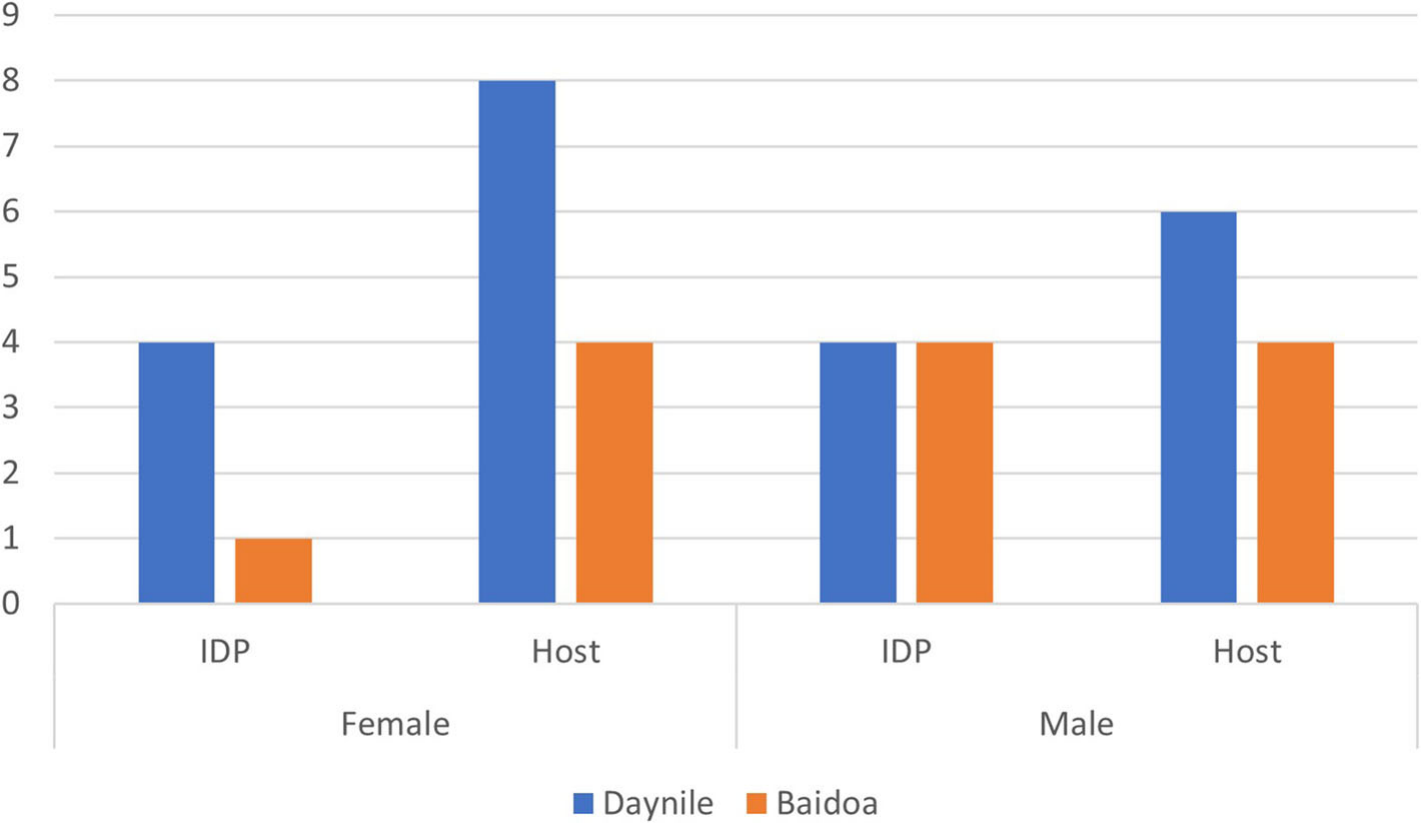
Gender distribution of participants across the study sites

Study participants were recruited from the self-selected AVF ‘Imaqal’ interactive media programme participant database, a gender equality and social inclusion focused communications programme in South Central Somalia and Puntland, through which they previously received radio and SMS messages on COVID-19 symptoms and measures. Participants were informed in Somali language about the voluntary bases of participation at the start of data collection through a consent flow protocol. Based on available demographic data from the Imaqal participant database, we sampled and invited 121 people to participate, of which 51 opted in. Following the introductory messages, 13 participants did not further engage, while we dismissed three conversations for analysis as the participants were underage. The final sample used for analysis consisted of 35 conversations, of which 17 were female − 12 in Mogadishu (4 IDP), 5 in Baidoa (1 IDP), and 18 male participants − 10 in Mogadishu (4 IDP), 8 in Baidoa (4 IDP) (Fig. 1). The youngest age group was considerably more responsive, with 30 participants in the age group 18–35, 3 participants between 36 and 54 and only 1 over 55 years old, which is reflective of a population where only 42 % falls within the working age group (15–64) [32].

### Data collection

The qualitative study was conducted over three months, with text messages sent out from Monday – Friday, through the online 1-to-1 SMS conversations interface in ‘Katikati’. Messages were designed in English by DB and translated into Somali by ZS, checked by AVF colleagues. Responses were collected by DB and translated by ZS. A total of 1563 messages was sent, out of which 745 response messages were received, including recruitment and exit messages. Individual conversations lasted 2–9 weeks, with the most active conversations sharing 100 messages back and forth. Engagement among participants was varied, and decreased significantly when government restrictions were lifted throughout July and August.

### Analysis

Responses were collected in a database, coded using a grounded theory to establish the main emerging themes as discussed by participants [33]. We conducted a narrative analysis, triangulating data with published data from quantitative surveys and other literature.

## Results

### Health and economic impact

The majority of participants (*n* = 29) reported being affected either directly or indirectly by COVID-19. Two participants reported contracting the disease themselves and/or their family members, while others had seen people with symptoms in their community (*n* = 7). Two participants lost relatives due to the disease, both in Somalia and among the Diaspora in the United Kingdom.

“Yes, it has affected us in a big way, we have lost some of our relatives, I have personally contracted the virus and my children also but we have survived, we have used some herbal medicine. It has also affected my daily income and the economy in general” (male, age 37, Daynile, host population).

Besides the two personal cases, which were confirmed by testing according to the participants, only one other participant mentioned family members receiving a COVID-19 test. Host population participants reported that free testing is available in Martini hospital in Mogadishu and Central Hospital Baidoa, while others reported a lack of testing facilities, or a refusal to test. As there is no treatment for COVID-19, people opted for traditional methods such as ginger and lemon, as well as self-isolation while displaying disease symptoms. Quarantine is considered difficult however for people in large households living in small accommodations.

Notably, four participants did not feel affected by the disease at all, in terms of health or otherwise. All of these were female members of the host population between 18 and 24 years old, and reported not knowing of any COVID-19 cases in their family or communities. Two 25 year old male participants initially claimed not to be affected by COVID-19, however did see a deterioration of the economic situation in their community, while another 25 year old male put COVID-19 into perspective, as he perceived the biggest health risk to be the lack of medication for other endemic diseases. Several participants (*n* = 3) point out that the economy and/or their lack of income is a bigger concern.

‘I don’t think there is coronavirus in the community; there a lot of changes [in] health, economy and community finance; one of the health challenges is that the hospitals don’t have enough medicine; [for] many diseases like Hepatitis typhoid and other diseases’ (male, age 25, Baidoa, host population).

‘It has impacted me in a big way financially; it has affected the market, everything has become expensive, food in shops are expensive; I have not received any kind of assistance from anyone and nobody asked me if I need help; I am buying cheap items nowadays’ (male, age 18, Daynile, host population).

By far the largest impact of the pandemic to participants was a result of government-imposed curfews, lockdowns and travel restrictions, unsurprising in a population highly dependent on daily wage labour. Ten people responded that their education was affected, while sixteen people lost income and/or their job, and others noticed that the disease had more generally affected their community. The level to which people were affected economically, depended on the type of livelihood or other daily activity such as education or household responsibilities.

‘Coronavirus has had a big impact on me, it has affected my economy and my education; it has reduced movement and transport, nothing is leaving or entering the city; because of that the economy has gone down’ (female, age 20, Baidoa, host population).

‘I was a teacher when the virus reached the country schools were closed and that is how my salary and income stopped and now I have joined the IDPs of Baidoa’ (male, age 25, Baidoa, IDP).

Income of - internal displaced - people selling goods or services on the market (*n *= 2), and restaurant staff (*n* = 2) decreased, or lost altogether, as they received fewer customers due to social distancing and curfews, while those selling products on the streets (*n* = 2 female, male IDP) and operating ‘Bajaaj’ taxis faced limitations to their business due to movement restrictions. The closure of schools affected two teachers who lost their jobs, as well as students. Meanwhile, some - host population - participants mentioned that prices increased (*n* = 2), while remittances received from the Diaspora decreased. One male IDP could no longer send his children to school due to the loss in income.

‘Coronavirus has greatly affected my life; I have lost the finances which I used to pay my bills like electricity, water and children’s education, the reality right now is that I have stopped my children from going to school and Madrasas’ (male, age 32, Baidoa, IDP).

‘It has affected the economy and the consumption of goods in the market is very low, this is because the remittance money people used to receive from their relatives in the diaspora have stopped due coronavirus’ (male, age 18, Daynile, host population).

### Responses and coping mechanisms

Coping mechanisms depended on the remaining income and livelihood opportunities, available savings, (extended) family support, religion, and the availability of and trust in health services. Some participants reported buying less, other using savings or relying on family, as they faced a reduction or loss of income. One teacher manages to survive as he lives with his family, while another participant moved in with her mother-in-law. While one participant got newly displaced, another young IDP returned from Daynile to Marka in April to start up a new business.

‘my grandfather, grandmother and uncle have died because of it, and they died in Mogadishu and England, those are the people who I depended on for finances; I [now] live with the mother of my ex-husband so that I can afford milk for my infant child’ (female, age 27, Daynile, host population).

Some participants used free healthcare facilities for other diseases, while others used private or NGO hospitals, although for some the costs of healthcare facilities are too high.

‘appointment with the doctor costs money, testing costs money, medicine costs money and everything costs money and the people cannot afford it’ (female, age 33, Daynile, IDP).

Participants believe that healthcare services have been greatly affected, mentioning a lack of personal protective equipment (PPE) and medication, as well as a lack of doctors. The lack of equipment and medication available to doctors was noted by one participant as causing reluctance to treat other health issues in hospitals during COVID-19.

‘[community leaders] started sharing health guidelines similar to those of health experts, we expected them to set up testing centers where people who think they have COVID-19 can go and get tested; it has affected [access to healthcare] in a big way; things like medicine and lack of hospital equipment, doctors are protecting themselves against this virus and are afraid to engage with the sick people’ (male, age 18, Daynile, host population).

### Awareness and religion

People across age/ gender groups received RCCE messaging through media channels, primarily radio, social media, SMS, door-to-door visits by officials, and friends. As our participants previously participated in an AVF COVID-19 RCCE interactive radio-SMS project, all had a basic level of awareness, but their uptake differed, while some expressed concern about corruption and misinformation.

‘Somalis are difficult people to talk and they don’t the risks of COVID-19, coronavirus is real we need to be careful’ (female, age 25, Daynile, host population).

‘they share it [through] SMS, radio and people who visit houses to create awareness; I have heard from the ministry of health of the South West State of Somalia’ (female, age 20, Baidoa, host population).

‘I believe COVID-19 is a virus that exists but it does not exist in Somalia, the media and government agencies just want to take contracts for coronavirus’ (male, age 20, Daynile, IDP).

Most participants knew the COVID-19 symptoms. Two female participants - both IDPs - mentioned that elderly and/ or people with underlying conditions - high blood pressure and diabetes - are more at risk of COVID-19. Two other female participants are aware that the disease can spread by asymptomatic people through air/ germs, learned in school about other diseases.

‘the things that are dangerous are places where there is a crowd like hotels, marriage ceremonies and restaurants; we put effort on hygiene like washing our hands, we avoid places where there are many people, we give extra caution to elder people; I wear a face mask and the reason is this virus spreads in the air, so it’s good to protect yourself against this dangerous disease’ (female, age 18, Daynile, IDP).

‘The things that puts people at risk are lack of hygiene, people with pre-existing conditions like blood pressure and diabetes and also crowded places; I protect myself by wearing face masks, I wear gloves on my hands and distance myself from other people; I take this measures every time since the dangers of coronavirus were mentioned; awareness creation is done day and night through radio stations and other media which has led to people to learn a lot about this virus because it’s something we have never heard of before’ (female, age 30, Daynile, IDP).

Most participants were aware of the government guidelines, including social/ physical distancing (*n* = 8), avoid crowded places (*n* = 5), the need to practice hygiene (*n* = 5) including washing hands, using personal protective equipment such as facemasks (*n* = 5) and gloves (*n* = 1), although three male participants found it hard to find, buy and/or wear these. Two male participants - both host community members - mentioned sharing the guidelines within their household and/or community.

‘It’s hard to get a mask and other things that prevent against the spread of coronavirus and some of the people cannot afford it; first there is no specific place where masks are sold, I see them in health centers and few people who work for NGO’s that given the masks to distribute to the community but instead they are selling the masks for 0.5 dollar’ (male, age 22, Baidoa, IDP).

Religion played an important role in people’s lived experience during COVID-19, across all age groups, genders and population types. People put their faith in the help of God to prevent them or the community from getting infected, end the pandemic, or revive the economy. While most were aware that the disease does not discriminate based on religion, some of those who were not personally affected attributed this to their religion, while others believed Somalia was spared as a Muslim country. One participant believed ‘going against the religion’ posed a potential infection risk.

‘The community are assisted; by God, because we are a Muslim community; coronavirus has ended; because God has ended it’ (female, age 24, Daynile, host population).

‘God has protected me from it but I am not sure about what I would do; because it is hard to quarantine when you are at home and people live with you’ (female, age 20, Baidoa, host population).

‘I don’t think there is coronavirus/ COVID-19 in the community thanks to God for ending this virus; I think it has ended because the people have started behaving like they used to before corona and they have forgotten about corona and how it has affected the world’ (male, age 22, Baidoa, IDP).

## Discussion

### Prevention and disease

The risk of disease is higher among populations lacking access to resources and services, with socio-economic status influenced by education and occupation [30]. IDPs are at particularly high risk, as they often live in precarious conditions, are dependent on external assistance and often unable to take measures to prevent disease. As the participants in our research self-selected from a cohort which previously participated in AVF COVID-19 RCCE interactive radio-SMS programming, they were generally aware of COVID-19 symptoms, increased vulnerabilities and protective measures. Already in March, a survey conducted by the Norwegian Refugee Council (NRC) found that three-quarters of participants were familiar with vulnerabilities to and the symptoms of the disease, while 58 % of people knew about protective measures, which might be attributed to the rapid and drastic government measures early on in the pandemic, supported by response agencies already present in-country due to the ongoing complex humanitarian emergency [34].

In our study, most participants were aware of the risk of infection through contact with an infected person, while one participant was aware of asymptomatic disease transmission. These findings reflect a survey conducted by Save the Children, which found that two-thirds of participants believed the disease is transferred through direct contact with an infected person, 58 % knows it is airborne, while 54 % mentioned contaminated surfaces and 49 % through droplets [35].

The lack of sanitation and handwashing facilities is a major risk for COVID-19 transmission, especially in Somalia where only 42 % has access to fresh water and 65 % to improved water sources [32], and 50 % lacks access to soap [36]. Online surveys measuring the adherence to government recommendations of five main preventive measures - physical distancing, face mask use, hand hygiene, mouth covering when coughing/sneezing, and avoidance of touching the face - found that adherence depended on gender, being a healthcare worker, obtaining information from official sources and level of education [6]. High uptake of preventive measures was reported by Alawa et al. [18], with over 77 % of participants taking at least one protective measure. Some participants in our study identified challenges with following the measures however, in particular related to inadequate shelter.

Even though official COVID-19 case numbers have remained low, in our small sample we found a number of people who had experienced COVID-19 themselves, or among family members. A nationally representative household survey found that almost a third of participants experienced at least one COVID-19 symptom, although only 12 % of these were tested, due to a lack of the availability or access testing facilities due to costs and transportation issues [36]. While a third of people used traditional remedies such as ginger, 30 % of the people experiencing symptoms denied it could be COVID-19 [36]. Some reports indicate that stigma and the lack of quality healthcare limits the number of people coming forward with symptoms for tests [37].

Up to 60 % of the Somali population does not have access to health services, while less than one-fifth of facilities have adequate equipment and medical supplies [12], noted as a major concern by several of our participants. Distance, medical fees, the lack of medication and personnel, and discrimination affect access to medical care [32, 34]. Lacking access to healthcare facilities in non-pandemic times, due to COVID-19 people have been even more reluctant to visit clinics for primary healthcare visits and vaccinations, resulting in untreated infectious diseases [38]. Walker et al. [8] warn that ‘mitigation strategies that slow but do not interrupt transmission will still lead to COVID-19 epidemics rapidly overwhelming health systems’, while the reduction in healthcare seeking during the pandemic might also lead to excess morbidity and mortality.

### Work and income

As a country dependent on imported goods, the closure of airports and cancellation of flights had a direct effect on market prices, as reported by our participants and confirmed by data from FEWS NET [39, 40]. While the government has tried to offset the price increases by temporary tax exemptions on basic commodities, this had limited effect as retailers started stockpiling [39]. Surveys showed that the increase in prices led to 34 % participants unable to buy essential food items [18], and up to 70 % started skipping meals [34].

As reported by our participants, international responses to COVID-19 further affected the Diaspora and remittances, which decreased by about 36 % in April [40]. By September, at least half of people surveyed nationwide reported receiving less remittances [34, 36].

In the March NRC survey, a vast majority of participants mentioned the negative impact of the closure of schools and madrasas (92 %), market inflation (67 %), community panic (64 %) and work stoppages (60 %). In April AVF reported that 8.1 % of participants was unable to go to work and 16.8 % lost their jobs, while by September surveys showed under- and unemployment now affected over two-thirds of the population, resulting in substantial decreases in household income, with IDPs disproportionally affected [34, 36, 41], reflected in our study. The closure of schools affected over one million children in Somalia according to the UN [17], reflected in our findings among students and their parents. This in turn has longer term health implications, as education is an important indicator not only for socio-economic status, but also influences disease prevention knowledge and uptake of RCCE messaging [30].

### Communication and trust

 Participants received awareness messages and guidance through (social) media, radio, SMS and in-person conversations with community leaders and friends. As most households in Somalia have access to a mobile phone with radio [32], surveys conducted by NRC and Save the Children in March and April 2020, showed that the majority of participants used radio as primary source of RCCE messaging, followed by social media or phone, television, and local authorities, community leaders, neighbours or other community members [34, 35], although awareness among women and IDPs remained relatively low [18, 42]. Religious leaders and radio shows are considered the most trustworthy sources of information, together with health officials and aid workers [18, 42], with some organizations engaging religious leaders to share important messages on COVID-19 and safe practices on radio shows [41].

While only 12.5 % cited the importance of prayer or other religious practices in the NRC survey, in our study most participants refer to religion in relation to protection or salvation from the pandemic. Other surveys show that for at least a third of the population religious practices and guidance is important [18, 41]. Save the Children’s RCCE assessment found that some believed that only religion could protect them against the virus [35].

We found that participants were sometimes misinformed about disease symptoms, but more importantly some lacked trust in authorities, healthcare services and humanitarian responders. IOM [14] points out the importance of ‘adopting an inclusive and integrated approach to counter misinformation and stigma’. A study by AVF in April 2020 found that over 10 % of participants reported stigma or misinformation, in particular IDPs outside of Benadir [41]. WHO and partners developed the RCCE (Risk Communication and Community Engagement) guidelines in response to the Ebola epidemic, to reduce stigmatization, enable prevention and access to services, by promoting a two-way communication strategy [35].

### Coronavirus is over

Most COVID-19 restrictions imposed by the Somali authorities were lifted and international flights were reinstated by early August, reflected in our study as we started receiving messages across the cohort claiming that the number of COVID-19 cases was ‘going down’ and that ‘coronavirus is over’.

One participant described the pandemic in Somalia in two ‘phases’: while the initial government measures and restrictions raised the awareness of the disease and protection measures, by the end of July restrictions were lifted and life returned to ‘normal’, and schools reopened by mid-August. Ahmed et al. [6] showed that adherence to government guidance decreased between April and July, even though twice as many people reported flu-like symptoms by July.

Participants who did not experience a COVID-19 case in their household or community believed the disease had ‘ended’ or never affected Somalia at all. One male participant claimed to have heard from friends that a vaccine was found. By mid-August, once restrictions were lifted, several participants disengaged from the conversation as they believed the disease was no longer impacting the country. Only three participants remained cautious and warned that Somalis should remain vigilant and keep taking the disease risk seriously.

### Study limitations

 While the remote method provided an opportunity to engage with participants during lockdown and access restrictions, the method is dependent on participants’ access to mobile phones and electricity, and ability to use SMS, potentially resulting in a relatively young cohort of respondents. The method does not allow for participant verification, and it is therefore possible that several people engaged on the same mobile phone over time. The study is not meant to provide a representative sample for extrapolation onto a wider population. Rather the study approach aims to deliver timely contextually-grounded qualitative findings from deeply personal experiences that may in turn guide further research or inform response decision-making.

## Conclusions

The COVID-19 pandemic has greatly affected the people of Somalia, their resilience depending primarily on health and economic outcomes of the pandemic, as well as the level of awareness of the disease and preventive measures. We found multiple individuals and households reporting COVID-19 symptoms, disease and community transmission. People whose health was affected by the disease remained predictably more careful in their responses to the pandemic. However, following a global trend, with the lifting of lockdown in August, general resistance against measures such as facemasks and social distancing increased (WHO, July 2020). While the confirmed number of cases in Somalia is relatively low, lack of availability of and access to testing facilities, combined with denial, stigma, and a lack of trust in health services, the unofficial disease burden remains unclear. The resurgence of COVID-19 in countries which managed to retain low caseloads during the ‘first wave’ should count as a warning against complacency.

Perhaps more importantly, the pandemic has affected people’s vulnerability to further shocks, affecting socio-economic health determinants such as livelihoods, remittances and household income – reflected in the COVID-19 related displacement of two of our participants. Authorities have duplicated international policy responses, including physical isolation and movement restrictions, sometimes exacerbating local vulnerabilities by limiting access to food and medical supplies, discounting local experiences and community responses. While these issues are not unique to Somalia, the socio-economic risks of rigid public health response are much higher in this conflict-affected context, but less of a focus of authorities and supporting agencies. For an effective response planning, more data is required beyond the localized, selective COVID-19 surveys conducted so far, and needs to include qualitative contextualized information beyond basic demographic, economic and health data, including spatial and social data, and citizen-generated knowledge[20]. There is a need for a multidisciplinary, intersectoral, inclusive response, focused on social and economic interventions, as well as public health control measures. Continuous awareness raising, improved free and accessible health services, income support and a return to- or alternative education need to be addressed to deal with the ongoing COVID-19 pandemic.

## Data Availability

All data generated or analysed during this study are included in this published article.

## Abbreviations

AVF: Africa’s Voices Foundation
CCCM: Camp Coordination and Management
DTM: Displacement Tracking Matrix
IDP: Internally Displaced Persons
IOM: International Organization for Migration
LMIS: Low and middle-income settings
MoH: Ministry of Health
NRC: Norwegian Refugee Council
PPE: Personal Protective Equipment
RCCE: Risk Communication and Community Engagement
UK: United Kingdom
UN: United Nations
WB: World Bank
WHO: World Health Organization

## References

[1] Heritage Institute. State of Somalia. 2020. Available from: https://reliefweb.int/sites/reliefweb.int/files/resources/SOS-REPORT-2020-Final-2.pdf.

[2] World Bank. Somalia Economic Update, Fifth Edition: Impact of COVID-19: Policies to Manage the Crisis and Strengthen Economic Recovery. World Bank; 2020. Available from: https://openknowledge.worldbank.org/bitstream/handle/10986/34239/Somalia-Economic-Update-Impact-of-COVID-19-Policies-to-Manage-the-Crisis-and-Strengthen-Economic-Recovery.pdf?sequence=6&isAllowed=y. [cited 2 May 2021]

[3] Heritage Institute. The Economic Impacts of COVID-19 on Somalia. 2020 Nov. Available from: https://heritageinstitute.org/wp-content/uploads/2021/01/The-economic-impacts-of-Covid-19-on-Somalia.pdf.

[4] Gele AA, Ahmed MY, Kour P, Moallim S, Salad AM, Kumar B. Beneficiaries of conflict: a qualitative study of people’s trust in the private health care system in Mogadishu, Somalia. Risk Manag Health Policy. 2017;10:127–35. 10.2147/RMHP.S136170. 10.2147/RMHP.S136170 PMC5548277 28831275

[5] WHO. Key Country Indicators. Geneva: World Health Organization; Available from: https://apps.who.int/gho/data/node.cco.latest?lang=en.

[6] Ahmed MAM, Siewe Fodjo JN, Gele AA, Farah AA, Osman S, Guled IA, et al. COVID-19 in Somalia: Adherence to Preventive Measures and Evolution of the Disease Burden. Pathogens. 2020;9(9):735. 10.3390/pathogens9090735. 10.3390/pathogens9090735 PMC7560173 32899931

[7] Alwan NA (2020). Surveillance is underestimating the burden of the COVID-19 pandemic. Lancet.

[8] Walker PGT, Whittaker C, Watson OJ, Baguelin M, Winskill P, Hamlet A, et al. The impact of COVID-19 and strategies for mitigation and suppression in low- and middle-income countries. Science. 2020;369. 10.1126/science.abc0035. 10.1126/science.abc0035 PMC7292504 32532802

[9] Davies NG, Klepac P, Liu Y, Prem K, Jit M, Eggo RM (2020). Age-dependent effects in the transmission and control of COVID-19 epidemics. Nat Med.

[10] Truelove S, Abrahim O, Altare C, Lauer SA, Grantz KH, Azman AS, et al. The potential impact of COVID-19 in refugee camps in Bangladesh and beyond: A modeling study. Parmar P, editor. PLOS Med. 2020;17(6):e1003144. 10.1371/journal.pmed.1003144. 10.1371/journal.pmed.1003144 PMC7297408 32544156

[11] WHO. COVID-19 Situation Report - Somalia. Geneva: United Nations; 2020 Oct.

[12] Ministry of Health and Human Services (2020). National contingency plan for preparedness and response to the coronavirus disease 2019 (COVID-19).

[13] WHO. Coronavirus Disease (COVID-19) Situation Report. Geneva: United Nations; 2020.

[14] IOM. Preparedness and Response Plan COVID-19. Geneva: United Nations; 2020.

[15] CCCM. RCCE COVID-19 Response by CCCM Cluster Partners as of 02 June 2020. Geneva: CCCM; 2020.

[16] WHO. COVID-19 Situation Report - Somalia. United Nations; 2020 Aug.

[17] UN. COVID-19 Response in Somalia 2020. Available from: https://covid19som-ochasom.hub.arcgis.com/. [cited 30 Sep 2020]

[18] Alawa J, Walz LA, Al-Ali S, Wiles E, Harle N, Abdullahi AM, et al. Knowledge and Perception of COVID-19, Prevalence of Pre-Existing Conditions, and Access to Essential Resources and Health Services in Somali IDP Camps. 2020. Available from: 10.1101/2020.08.17.20176271. [cited 6 Jan 2021]. PMC8245279 34187818

[19] Kinyoki DK, Moloney GM, Uthman OA, Kandala N-B, Odundo EO, Noor AM, et al. Conflict in Somalia: impact on child undernutrition. BMJ Glob Health. 2017;2(2). 10.1136/bmjgh-2016-000262. 10.1136/bmjgh-2016-000262 PMC5621625 28966793

[20] Wilkinson A, Ali H, Bedford J, Boonyabancha S, Connolly C, Conteh A (2020). Local response in health emergencies: key considerations for addressing the COVID-19 pandemic in informal urban settlements. Environ Urban.

[21] NRC. Downward Spiral: the economic impact of Covid-19 on refugees and displaced people. Norwegian Refugee Council; 2020.

[22] OCHA. Humanitarian Response Plan Somalia - COVID-19 Revision. New York: United Nations; 2020.

[23] UNFPA, Baidoa. COVID-19 Vulnerability Mapping by Risk Factors. New York: United Nations; 2020.

[24] Kamal A-HM, Huda N, Dell CA, Hossain SZ, Ahmed SS (2020). Translational Strategies to Control and Prevent the Spread of COVID-19 in the Rohingya Refugee Camps in Bangladesh. Glob Biosecurity.

[25] WHO. World Health Organization Humanitarian Response Plans in 2015.

[26] UNHCR. Somalia. COVID-19 Response. Geneva: United Nations; 2020.

[27] Scoones I, Jones K, Lo Iacono G, Redding DW, Wilkinson A, Wood JLN. Integrative modelling for One Health: pattern, process and participation. Philos Trans R Soc B Biol Sci. 2017;372(1725):20160164. https://doi-org.ezp.lib.cam.ac.uk/10.1098/rstb.2016.0164. 10.1098/rstb.2016.0164 PMC5468689 28584172

[28] Du RY, Stanaway JD, Hotez PJ. Could violent conflict derail the London Declaration on NTDs? PLoS Negl Trop Dis. 2018;12(4). 10.1371/journal.pntd.0006136. 10.1371/journal.pntd.0006136 PMC5908062 29672514

[29] Gayer M, Legros D, Formenty P, Connolly MA (2007). Conflict and Emerging Infectious Diseases. Emerg Infect Dis.

[30] Solar O, Irwin A. A conceptual framework for action on the social determinants of health: debates, policy & practice, case studies. 2010. Available from: http://apps.who.int/iris/bitstream/10665/44489/1/9789241500852_eng.pdf. [cited 2021 Jan 6]

[31] IOM. COVID-19 IDP Site Risk Map Version 2. Geneva: IOM; 2020.

[32] UNFPA. The Somali Health and Demographic Survey 2020. New York: United Nations; 2020.

[33] Strauss A, Corbin J, Denzin NK, Lincoln YS (1994). Grounded theory methodology: an overview. Handbook of Qualitative Research.

[34] NRC. A cough that kills people: Views on Covid-19 from Somalia’s displacement-affected communities. Geneva: Norwegian Refugee Council; 2020.

[35] Save the Children. RCCE Somalia COVID19 Rapid Assessment Survey Report (April 2020). London: Save the Children; 2020.

[36] NEXUS. The significant impacts of COVID-19 on the livelihoods and health of Somali communities: Findings from a nationally representative household survey. Mogadishu: Federal Government of Somalia; 2020.

[37] Jerving S. Stigma. and weak systems hamper the Somali COVID-19 response. Washington: Devex; 2020. Available from: https://www.devex.com/news/stigma-and-weak-systems-hamper-the-somali-covid-19-response-97895. [cited 2020 Sep 30].

[38] ICRC. Somalia. Decline in primary health care visits and childhood vaccinations during COVID-19. Geneva: International Committee of the Red Cross; 2020. Available from: https://www.icrc.org/en/document/somalia-sharp-decline-primary-health-care-visits-and-childhood-vaccinations-during-covid-19. [cited 2020 Sep 30].

[39] FEWS NET. Somalia Price Bulletin. Washington: Famine Early Warning System; 2020.

[40] FEWS NET. Food Security Outlook Update August 2020. Washington: Famine Early Warning System; 2020.

[41] Srinivasan S. Religion, rumour and right practice: Somali views in the early days of COVID19. Nairobi: 2020.

[42] CCCM. COVID-19 response - assessment on risk communication and community engagement in IDP sites. Mogadishu: CCCM Cluster and DTM; 2020.

